# Effectiveness of flossing loops in the control of the gingival health

**DOI:** 10.4317/jced.53858

**Published:** 2017-06-01

**Authors:** Francisco Azcarate-Velázquez, Roberto Garrido-Serrano, Gabriel Castillo-Dalí, María-Angeles Serrera-Figallo, Alfonso Gañán-Calvo, Daniel Torres-Lagares

**Affiliations:** 1 Master in Oral Surgery. Department of Dentistry. Faculty of Dentistry. University of Seville, Spain; 2 Department of Aerospace Engineering and Fluid Mechanics. University of Seville, Spain

## Abstract

**Background:**

One of the key factor in the good condition of periodontal tissues is their daily hygiene. Oral hygiene techniques such brushing and a good interdental hygiene by correct flossing are very important. The aim of this study is to compare the use of dental floss in a loop vs traditional floss in the control of Loe-Silness Gingival Index (IG), Turesky´s Plaque Index (IPT), Gingival Bleeding Index (IS) and the values of interleukin 6 (IL-6) and interleukin 8 (IL-8).

**Material and Methods:**

A comparative study of 40 patients in which each patient was his own control, using during 45 days each one of the devices (new loop floss and conventional floss) of interdental hygiene analysed. Data for Loe-Silness Gingival Index (IG), Turesky´s Plaque Index (IPT), Gingival Bleeding Index (IS) and the values of interleukin 6 (IL-6) and interleukin 8 (IL-8)were collected and measured in every visit for every type of interdental hygiene device.

**Results:**

Our data indicates that the rate of Turesky´s Plaque Index presented statistically significant differences between groups (loop: 1.66 ± 0.8; traditional: 1.12 ± 0.8; *p*<0.0001). The rest of the indices studied showed no statistically significant differences.

**Conclusions:**

The creation of new dental floss designs try to make their use easier and more sensitive, and plaque removal more effective. The loop design can facilitate interdental hygiene, reaching similar effectiveness than traditional floss, improving some indicators, such as Turesky´s Plaque Index.

** Key words:**Dental floss, bacterial plaque, loop floss, plaque index, periodontal diseases.

## Introduction

It is well known that the presence of bacterial plaque is associated with the development of gingival and periodontal disease (1). Good tooth brushing (TB) procedures are essential to achieve adequate results in plaque control and removal, and the use of dental floss in combination with TB has shown some evidences of improvement of interproximal plaque removal (2-5), in addition to the obvious help in interdental debris removal. However, current researches cast doubts on the benefits for floss on plaque and clinical parameters of gingivitis (6), pointing to a fundamental issue: the technique-sensitiveness of dental floss. In this regard, some findings point to a detrimental effect of the use of some kinds of floss under certain periodontal conditions (7).

Current general public exposure to information on general health guidelines allows paradigm shifts that, combined with self-care measures, can produce very significant positive global health impact. In the case of periodontal self-care (8), personal assessment of compliance with dental flossing towards plaque removal can be critical. To this end, nowadays there is a great amount of chemical methods in the market such as plaque erythrosine, malachite green, basic fuchsin, and other food dyes that are used to identified the presence of dental plaque.

Toothbrushes could be considered one of the main instruments for the removal of dental plaque. Those can be manual or electric. Some of the most well-known manual brushing techniques are the Bass technique or modified Stillman technique (9). In terms of effectiveness, electric toothbrushes with oscillatory / rotary head have a higher level of scientific evidence than manual toothbrushes, regarding to both plaque removal and gingivitis control. This not only comes to better plaque removal, but because the electric toothbrush makes brushing more comfortable and longer lasting (9).

As basic tools for removing interproximal plaque, dental floss has generally been used in string form, but also has been marketed in different forms, as attached to U-shaped supports, in order to facilitate its use. There is a wide range in the market: waxed or unwaxed, striplike or round section, rigid or soft and with different active ingredients such as fluoride, chlorhexidine or flavoring substances. Its final aim is to facilitate its movement over the proximal surfaces and cleaning of the small surface irregularities. A good quality string should stretch properly without fraying.

The use of interproximal brushes of different sizes, straight or angled, make the plaque removal easier (10,11), especially residues in posterior sectors. Also for interproximal cleaning are used interdental stimulators or cones (9).

Other instruments used are wooden wedges or toothpicks. These can be made of wood or plastic although its effectiveness is much discussed since their use, in some cases as in healthy patients, can be more harmful than beneficial (10).

Devices like oral irrigators use the pressure of the water against tissues and they are useful to remove food particles but not effective for plaque removal. They are only useful if used together with toothbrush and floss. Use can be made of chemicals to control plaque, for example, chlorhexidine (6,11,12).

For its general acceptance, low price, simplicity and robustness, dental floss has been considered a major device category for innovation and improvement. However, as noted above, its own simplicity, inherent technique-sensitiveness and consequent user low compliance leave little room for innovation and pose obvious clinical limitations.

The only room for certain innovation in dental floss to make it clinically attractive, while keeping the extreme simplicity and low price of the original device, would be in the direction of the improvement of user compliance. A basic mechanical conceptual approach in that direction would be restricting the large number of degrees of freedom allowed by the original device. In this paper, the most obvious, simple, robust, and yet never clinically studied innovation in this regard is considered: tying up the ends of a conventional floss to form a circle or closed loop. Major advantages foreseen over the original linear floss are: (i) drastic improvement of user compliance, (ii) easier handling, (iii) lower raw material and string waste, (iv) drastic improvement of string length use, (v) improved string hygiene, and (vi) improved plaque removal efficacy and periodontal condition.

**Figure 1:**
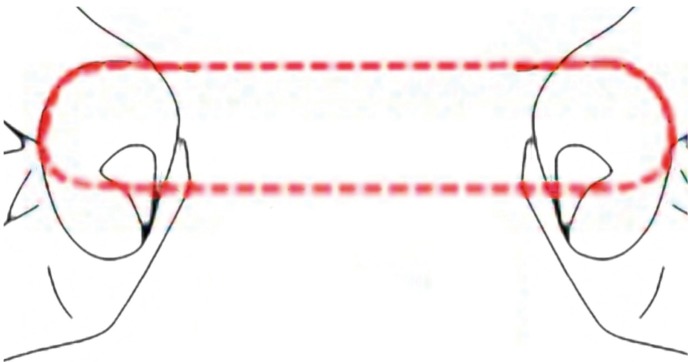
Looped dental floss. Design.

**Figure 2:**
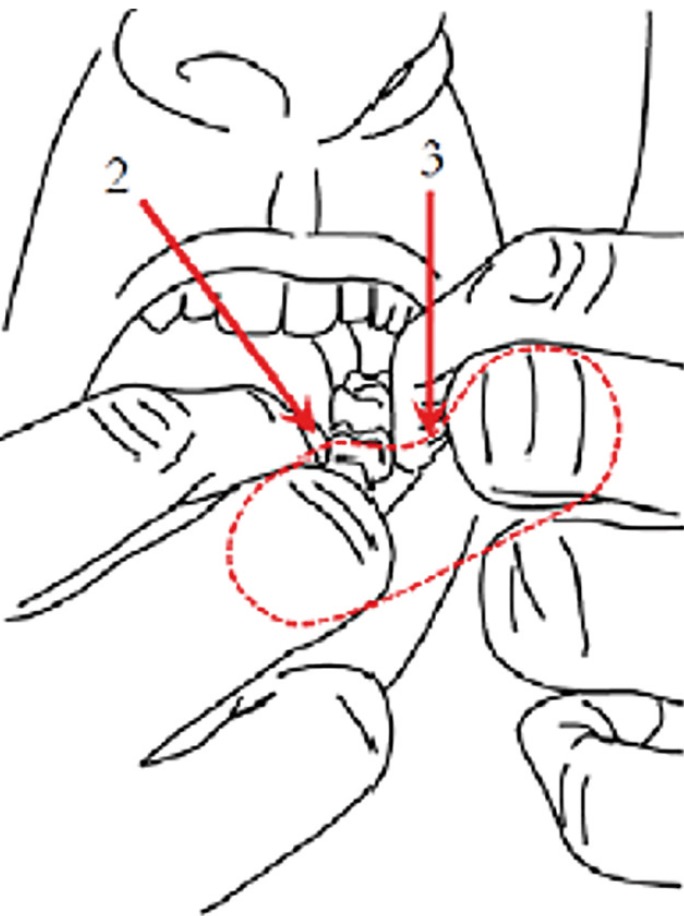
Biomechanics of looped dental floss.

Thus, the device studied in this article for interdental cleaning technique is a dental floss loop (Fig. 1). This loop is taken with several fingers to make it tense. Dental floss is passed through the interproximal spaces during usage, while it is rotated to allow the use a clean piece of floss in each interproximal space (Fig. 2). In this way, the efficiency of the use of floss material is maximum.

The current use of traditional dental floss demands a friction force with the skin of the fingers to keep it mechanically resistive to perpendicular forces associated to interproximal space insertion. Note that even if the user employs a generous extra floss length to make some loose loops around each finger, friction force is always present as the major mechanical component of the resistive forces. Such a result is highly variable depending on the nature of the skin, its moisture content, etc. In contrast, the looped dental floss configuration allows a tension in excess of the one of traditional floss without resorting to friction at all: even if the fingers are wet, acting like mechanical pulleys, the wire is kept under a tension force that is entirely imposed by the direct force that the user puts through his/her hands or arms. This restricts very significantly the variability felt by the user and provides a complete control on the loop. In addition, the loop provides the possibility of using its entire length in cleaning, only by turning the used floss progressively, ensuring a high value of effectiveness of the floss.

Besides, interleukin-6 is a multifunctional cytokine with biological activities such as B lymphocyte differentiation, T cells proliferation and stimulating the secretion of immunoglobulins (Ig) by B lymphocytes, stimulation of protein synthesis in acute phase and activation of the complement cascade (13). Particular importance is the ability of IL-6 to induce bone resorption, both by itself and in combination with other agents of bone resorption (14). These effects are developed especially in cases of bacterial infection and are therefore also of crucial importance in inflammatory periodontal diseases (15). Authors like Morrelli *et al.* in 2014 concluded that salivary levels of IL-1ra and IL-6 could be potential indicators of changes in probing depth and gingival inflammation-inducing (16).

IL-8 is a potent chemokine with function in the recruitment and activation of human granulocytes and mediation of inflammatory processes (17). It can be released from various cells, including monocytes / macrophages, lymphocytes, fibroblasts, endothelial cells, and epithelial cells (17,18). IL-8 plays an important role in the regulation of neutrophil function (19). Lütfioglu *et al.* in 2015 linked the increase in IL-8 in the presence of periodontal disease, this being higher in chronic aggressive periodontitis and gingivitis (20).

In our study, the benefit of using looped dental floss was studied in controlling dental plaque and gingivitis through the analysis of various clinical indicators and measuring the concentration of IL-6 and IL 8 in crevicular fluid.

## Material and Methods

Our clinical study was conducted at the clinic of the Faculty of Dentistry at the University of Seville after being approved by the Ethics Committee Research at Virgen del Rocio Teaching Hospitals in Seville, Spain. Research has been conducted in full accordance with the World Medical Association Declaration of Helsinki. We have obtained writted consent from all participants involved in your study (consent procedure was approved by the ethics committee).

The study began with the structuring of the study groups with a total of 40 randomized patients which should meet the following inclusion criteria: patients aged between 18 and 30 years without underlying pathology associated (hypertension, diabetes, endocrine pathology, among others), presence in the mouth of more than 20 teeth (excluding third molars) and absence of infectious disease or dental decay. Similarly, patients should not submit any of the exclusion criteria: injury or inability to perform brushing / use of loop, lack of mouth opening (<30 mm), presence of orthodontic appliances fixed, pregnancy, smoking or having periodontal disease.

**Figure 3:**
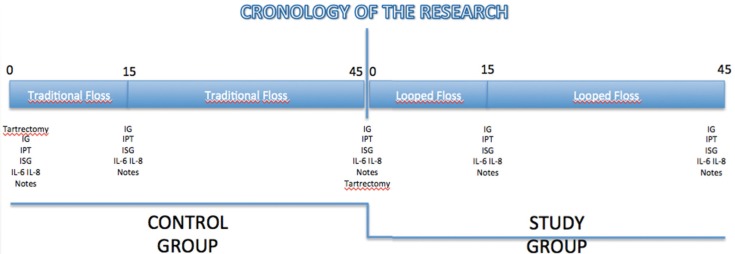
Chronology of the research.

All patients studied were students of the Degree in Dentistry at the University of Seville. They were randomized in a group (n = 40) that began with the use of conventional floss. This group underwent routine scaling and polish at baseline (T0), at 15 (T15) and 45 days (T45) days measurements and IL samples taken. After taking the samples T45 proceeded to make a new routine scaling all patients, and began using the method of looped dental floss. At 15 days (T60) and 45 (T90) the same operation takes data (Fig. 3) were repeated.

Clinical gingival index, Turesky´s plaque index, bleeding on probing index and the concentration of inflammatory markers interleukin 6 and 8, both present in the crevicular fluid were recorded and analyzed. 

Crevicular fluid samples were collected from the interdental areas in the lower lateral incisors with four two-centimeters long paper points previously sterilized. The soaking time for each patient was five seconds and immediately inserted into 0.5ml Eppendorf microtubes with 50 μl of saline at 4° C for preservation.

Transportation of samples to the biological laboratory was conducted in a cork refrigerator with thermal ice sheets, and frozen at -80° C on arrival to lab. Then we proceeded to analyze the concentration of interleukins (IL-6 and IL-8) detected in each sample by panels bioplex mark Luminex, which are based on binding between the direct immunofluorescence technique (DIF) and flow cytometry (FCM), through magnetized spheres labeled enzymatically with streptavidin, (which upon addition of its substrate subsequently generates a detectable and measurable coloured product), and marked with two specific antibodies (one for each Interleukin of study). The laboratory protocol began with a description of the detection method Bio-Plex Pro Assay. Reactive substances, calibration of “Luminex” reader and software were set up to proceed with the procedure running the assay (points 1-16). After this procedure, the proteins designated were analyzed for the prparation of the standars BioRAD - Bio-Plex Human Cancer Biomarkers Panel 2, 18 plex standars curves. This curve is used to interpolate the results of the cuantitiative measurement.

Thus, the presence of both these two interleukins are detected only and specifically for both their quantity and their concentration (pg / ml), simultaneously for both analytes.

The data were included in a data base SPP 15.0 for Windows, comparing data from both test systems using the U Mann-Whitney, with a level of statistical significance of *p* <0.05, using the software mentioned above.

## Results

From an initial sample of 40 individuals who met the inclusion criteria only 35 individuals completed the study. These five individuals who were lost during the study were discarded due to two causes: they took anti-inflammatory treatment for 90 days (two of them) or missed any of the visits (three of them). Among the 35 individuals who completed the study, there were five men and 30 women.

In reference to the Gingival Index (GI), there is a decrease between Visit 2 (0.95 ± 0.3) and visit 3 (0.87 ± 0.4) in control group as occurs between visit 2 group (1.00 ± 0.4) and visit 3 (0.98 ± 0.4) in the study group, the difference is not statistically significant (*p* = 0.69) ( Table 1).

**Table 1:**
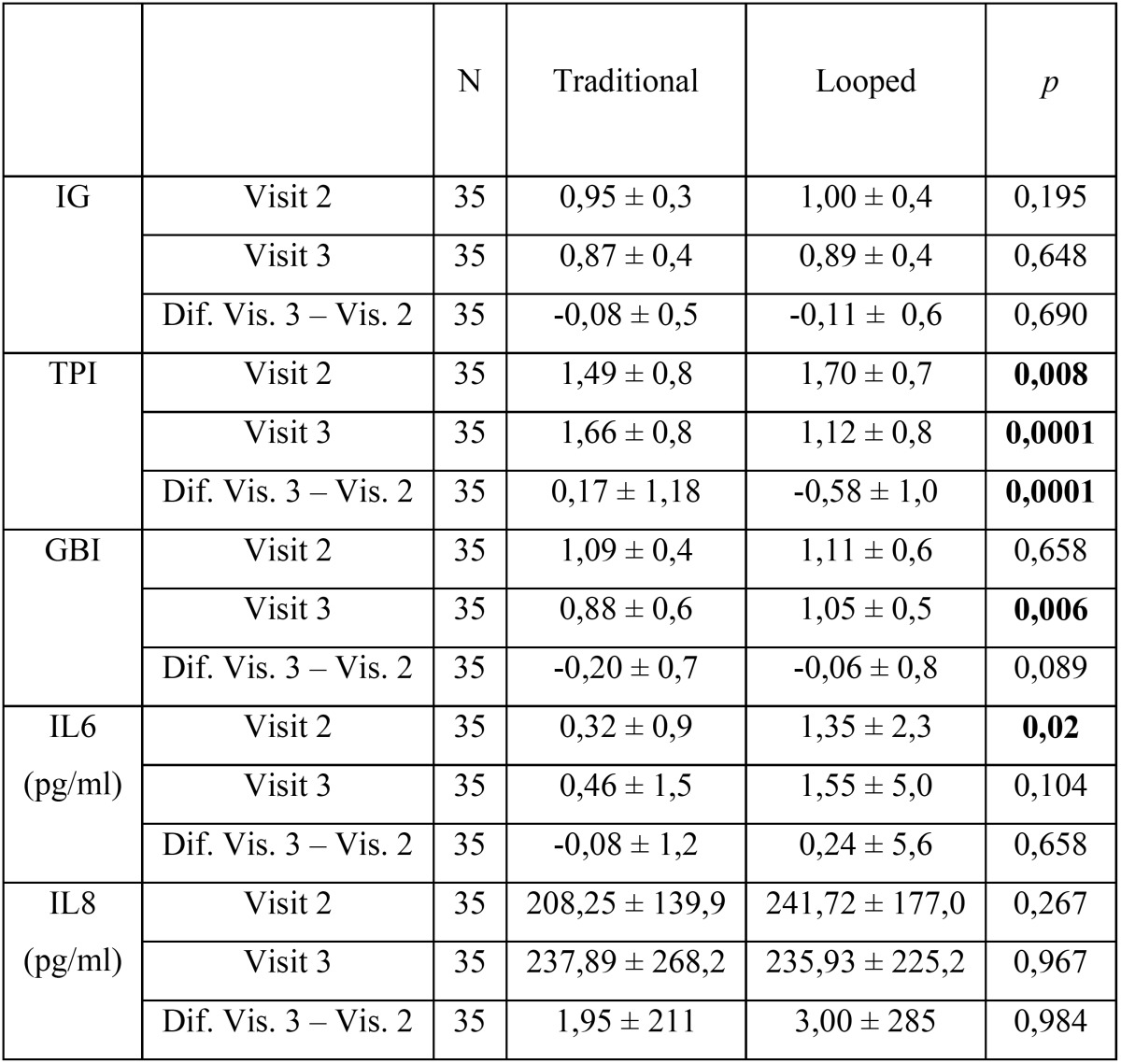
Results of IL6, IL8 Turesky´s Plaque Index, Gingival Bleeding Index.

When we analyze Turesky´s Plaque Index, we find that there is an increase in values between visit 2 (1.49 ± 0.8) and visit 3 (1.66 ± 0.8) in the control group, but the study group found a decrease between visit 2 (1.70 ± 0.7) and visit 3 (1.12 ± 0.8), the difference is statistically significant (*p* <0.05) ( Table 1).

Regarding to the Gingival Bleeding Index (ISG), there is a decrease between Visit 2 (1.09 ± 0.4) and visit 3 (0.88 ± 0.6) in the control group as occurs between visit 2 (1.11 ± 0.6) and visit 3 (1.05 ± 0.5) in the study group, the difference is not statistically significant (*p* = 0.089) ( Table 1).

Results for biochemical parameters like Interleukin 6 (IL-6) showed that there is a levelling-off between different values between visit 2 (0.32 ± 0.9) and Visit 3 (0.46 ± 1.5) in control group as occurs between visit 2 (1.35 ± 2.3) and visit 3 (1.55 ± 5.0) in the study group, the difference is not statistically significant (*p* = 0.658) ( Table 1).

In respect to Interleukin 8 (IL-8), there is a levelling-off between different values between visit 2 (139.9 ± 208.25) and Visit 3 (237.89 ± 268.2) in control group as occurs between visit 2 (241.72 ± 177.0) and visit 3 (235.93 ± 225.2) in the study group, the difference is not statistically significant (*p* = 0.984) ( Table 1).

## Discussion

Throughout history, many methods have been used for the prevention and treatment of periodontal disease. Typically, dental floss is used as a control method of interdental plaque. Its use requires a learning period which depends on various factors such as patient’s ability or patient´s oral health motivation.

In order to obtain the maximum levels interdental plaque removal, dental floss should be combined with a good brushing technique. That is why our study combines the use of brushing with dental floss (21,22).

Two techniques flossing were compared in this study, conventional floss and looped dental floss, analyzing their effectiveness in removing plaque.

Systematic review conducted by Chapple *et al.* in 2015 (23) supports the need to control dental plaque to maintain gingival health through electric toothbrushes. The use of floss is only recommended for those locations where you cannot use interdental toothbrushes without trauma. Therefore, according to this autor, the interpoximal toothbrushes would be the instrument of choice to maintain gingival health.

Salzer *et al.* in 2015 (24) evaluated the effectiveness of different instruments to control interdental plaque through a systematic review. They concluded that interproximal brushes were the most effective method and most of the studies did not demonstrate that the use of floss was effective. All devices help to control gingivitis. Our study evaluates the technique of using dental floss.

More contemporary studies such as Cronin *et al.* compared electric interdental cleaning devices, flosser, toothpick, and traditional dental floss with wax. They cncluded that the toothpick group had a higher plaque index compared to the others (25). These studies show that dental floss generally reduces the presence of plaque in a satisfactory manner without encountering difference between the different types.

Various studies show that the addition of excipients such as tetrasodium pyrophosphate podwer, due to its anti-plaque action, only lasts two hours after application (26). In our study we have evaluated the technique itself for the removal of plaque.

Terezhalmy *et al.* in 2008 compared the effectiveness in removing plaque from four different types of dental floss in their microstructure (three conventional silks: unwaxed, intertwined and resistant to breakage and floss in stick) concluding that there was no difference statistically significant between them although floss in stick removed more plaque (27). It showed that there was only statistically significant difference compared with brushing alone. In our study the type of floss material was not evaluated, only its presentation and minimal differences were found, hence both systems have a similar efficiency.

Shibly *et al.* in 2001 compared different presentations of dental floss. In this case they were compared conventional floss to “automatic power flosser”. No differences between the gingival index and plaque index were found (28).

Ciancio *et al.* compared two types of dental floss (waxed and expanded polytetrafluoroethylene) concluding that flossing generally improves plaque removal, it does not matter its presentation (29).

Mizutani *et al.* evaluated the oral health according bleeding, a questionnaire oral hygiene habits and self-efficacy scale hygiene. They concluded that there was correlation between good hygiene, effectiveness and college students (30). Individuals used in our study may be hypermotivated in their oral hygiene habits.

Some studies show that there is an increase in the concentration of IL-6 when we are faced with an episode of chronic (31) perio-dontitis or aggressive. In our study we do not have any of these case and for them the values of IL-6 were stable during the study. Morrelli *et al.* in 2014 concluded that salivary levels of IL-1 and IL-6 could be potential indicators of changes in probing depth when gingival inflammation (16) is induced. Compared with our study, we found no increase in probing depth or levels of IL-6. Other studies show that only the values for prostaglandin E2 and macrophage inflammatory protein (MIP) -1a are the only ones that increases and allow us to discern between health and gingivitis (32).

Some studies show significant changes in values in crevicular fluid when periodontal disease is in an advanced stage and / or aggressive (20) and even in cases of peri-implant pathology as perimplantitis (33), showing a positive correlation between probing depth and the presence of IL-8 (34).

In conclusion, the results of our study indicate that the use of looped dental floss is useful in removing dental plaque and gingivitis control. While most clinical indicators and IL6 and IL8 were levelled off between the two groups, a significant improvement in the rate of Turesky´s Plaque Index in the looped dental floss group was identified.
